# Larotinib in patients with advanced and previously treated esophageal squamous cell carcinoma with epidermal growth factor receptor overexpression or amplification: an open-label, multicenter phase 1b study

**DOI:** 10.1186/s12876-021-01982-4

**Published:** 2021-10-23

**Authors:** Rongrui Liu, Lianke Liu, Chuanhua Zhao, Yuxian Bai, Yulong Zheng, Shu Zhang, Ning Li, Jianwei Yang, Qingxia Fan, Xiuwen Wang, Shan Zeng, Yingjun Zhang, Weihong Zhang, Yulei Zhuang, Ning Kang, Yingzhi Jiang, Hongmei Sun, Jianming Xu

**Affiliations:** 1 Department of Oncology, The Fifth Medical Center of Chinese PLA General Hospital, No. 8 Dongda Avenue, Fengtai District, Beijing, 100071 China; 2 Department of Oncology, Jiangsu Province Hospital, Nanjing, China; 3 Academy of Military Medical Sciences, Academy of Military Sciences, Beijing, China; 4 Department of Internal Medicine, Harbin Medical University Cancer Hospital, Harbin, China; 5 Department of Medical Oncology, The First Affiliated Hospital, Zhejiang University School of Medicine, Hangzhou, China; 6 Department of Gastroenterology, Shandong Cancer Hospital, Jinan, China; 7 Department of Medical Oncology, Henan Cancer Hospital, Zhengzhou, China; 8 Department of Medical Oncology, Fujian Provincial Cancer Hospital, Fuzhou, China; 9 Department of Oncology, The First Affiliated Hospital of Zhengzhou University, Zhengzhou, China; 10 Department of Chemotherapy, Qilu Hospital of Shandong University, Jinan, China; 11 Department of Oncology, Xiangya Hospital Central South University, Changsha, China; 12 Sunshine Lake Pharma Co., Ltd, Dongguan, China; 13 Department of Oncology and Hematology, Jiamusi Tumor Tuberculosis Hospital, Jiamusi, China

**Keywords:** Larotinib, EGFR TKI, Esophageal squamous cell carcinoma, EGFR overexpression, EGFR amplification

## Abstract

**Background:**

Larotinib is a new first-generation epidermal growth factor receptor (EGFR) tyrosine kinase inhibitor. This open-label, phase 1b study is aimed to evaluate the efficacy, safety of larotinib in patients with advanced esophageal squamous cell carcinoma (ESCC) with EGFR overexpression or amplification pretreated with one or more system regimens, and to recommend an appropriate dose for its further study.

**Methods:**

Patients received larotinib orally at 3 doses (250, 300, 350 mg), once daily. Clinical response was evaluated every 8 weeks according to RECIST v1.1 criteria by both investigators and independent radiology review (IRC).

**Results:**

81 patients were enrolled. The investigator-assessed overall response rate (ORR) was 13.7% (10/73), all responses were observed in the 350 mg group of which ORR up to 20.0% (10/50), with 10 of them having EGFR overexpression and 4 having EGFR amplification. Per IRC assessment, ORR for all patients and 350 mg group were 13.9% (10/72) and 16.3% (8/50). In the 350 mg group, median overall survival (OS) and progression-free survival (PFS) were 8.0 (95% CI 4.9–10.2) months and 3.4 (95% CI 2.4–3.7) months, respectively. The most common treatment-related adverse events (TRAEs) were diarrhea, rash, and palmar-plantar erythrodysesthesia syndrome, elevated AST/ALT, vomiting, similarly with other EGFR TKIs.

**Conclusions:**

Larotinib demonstrated promising antitumor activity and manageable safety profiles in patients with pre-treated advanced ESCC with EGFR overexpression or amplification, especially at the dose of 350 mg, which showed better efficacy and acceptable safety. A phase 3 study is underway on 350 mg larotinib in ESCC patients with EGFR overexpression.

***Trial registration*:**

This trial was retrospectively registered on 25/03/2019, NCT03888092. https://clinicaltrials.gov/ct2/show/NCT03888092.

**Supplementary Information:**

The online version contains supplementary material available at 10.1186/s12876-021-01982-4.

## Background

Esophageal cancer is one of the most common malignant tumors, and is the 8^th^ leading cause of cancerous death worldwide, with an estimated 572,034 new cases and 508,585 deaths in 2018 [1]. Esophageal cancers are histologically classified as squamous cell carcinoma (SCC) and adenocarcinoma [2]. SCC is the most common histology in Asia, accounting for 90% of esophageal carcinoma in China [3]. Despite considerable improvements in the treatment of esophageal cancer, the prognosis remains poor, with a 5-year overall survival rate of 16.3–18.7% [4].

To date, platinum-based combination chemotherapy remains the standard first-line treatment for unresectable locally advanced, recurrent, or metastatic esophageal cancer. Although agents such as irinotecan [5–7], docetaxel [8, 9], and paclitaxel [10, 11] have shown single-agent activity, there was no standard treatment for patients who failed first-line therapy, until the approval of PD-1 inhibitors, such as pembrolizumab, nivolumab, and camrelizumab in recently years. Although these immune checkpoint inhibitors showed efficacy in ESCC patients, the therapeutic response was limited [12–14]. Additionally, there is no good choice for ESCC patients who failed immune therapy. Therefore, new treatment options are still urgently needed for this patient population.

Epidermal growth factor receptor (EGFR) was reported to be overexpressed in about 50% of ESCC in Asia [15–17]. In addition, the overexpression of EGFR was considered to be associated with prognosis in many cancers, including esophageal cancer [18]. In a retrospective study on 447 patients, the survival rate of patients with high EGFR expression is significantly lower than that of patients with low EGFR expression (*p* = 0.000), the 5-year survival rates were 18.2%, and 41.5%, respectively [19]. In addition to protein overexpression, EGFR gene amplification was observed in ESCC [16], which indicated anti-EGFR therapies might be appropriate for patients in ESCC. Several clinical studies of first-generation EGFR tyrosine kinase inhibitors (TKIs) have been conducted in esophageal cancer. Nearly a decade ago, Petty and et al. conducted [20] a placebo-controlled, randomized, phase III trial in patients with previously esophageal cancer (the COG study), which enrolled 449 patients, 50 of whom were diagnosed with ESCC. Unfortunately, the COG study showed limited efficacy, with an ORR of only 2.7% (6/224), median PFS of 1.57 months, and OS of 3.73 months. This finding was similar to a phase II trial that was conducted earlier in esophageal cancer treated with erlotinib [21]. Possible Reasons behind the failure of both gefitinib and erlotinib studies included: (1) The majority of subjects recruited were diagnosed with adenocarcinoma [20–22]. These studies [20–23] suggested that ESCC is more sensitive to EGFR TKIs than adenocarcinoma, which was also observed in the erlotinib Phase II trial in esophageal cancer, with 2 responses observed in 13 patients living with squamous carcinoma, and no responses observed in the 17 patients with adenocarcinoma. (2) No EGFR biomarker selection was conducted in these study populations. The recent study suggested that patients in ESCC with EGFR overexpression or amplification had a higher likelihood to benefit from anti-EGFR therapy [23]. Therefore, to explore the efficacy of larotinib in the potential benefit subjects, ESCC with overexpressed or amplified EGFR was mandatory in this study.

Larotinib is a first-generation EGFR TKI. Preclinical studies indicated high selectivity of larotinib for EGFR kinase and high concentrations of the drug in tumor tissue. The ratio of AUC in tumor and plasma was over 20 (Additional file 1), possibly suggesting an optimal risk–benefit ratio. In the dose-escalation phase 1a trial of larotinib (ChiCTR-OPC-15007153), we investigated the safety profile of larotinib at the doses from 50 to 400 mg/d in patients with advanced solid tumor without biomarker screening, there was no dose limited toxicity (DLT) observed, and 2 partial responses appeared at 220 mg/d and 350 mg/d, both occurred in non-small cell lung cancer patients. In addition, tumor reduction was also observed in ESCC patients even at 100 mg/d in this study [24].

Based on these data, and the urgent unmet need, we performed the present phase 1b clinical trial to further assess antitumor activity and safety of larotinib in 3 doses (250 mg, 300 mg, or 350 mg) in patients with pre-treated advanced ESCC with EGFR overexpression or amplification (ClinicalTrials.gov identifier NCT03888092).

## Methods

### Study design and patients

This was an open-label, multi-center, phase 1b study, designed to evaluate the antitumor activity and safety of larotinib in patients with pre-treated advanced ESCC with EGFR overexpression or amplification. The protocol has been revised twice (Additional file 2). The protocol and all amendments were approved by all participating institutions. The study was conducted following the Declaration of Helsinki and International Good Clinical Practice Guidelines. All patients provided written informed consent.

For this phase 1b open-label trial, patients were screened from 13 study centers in China. The main eligibility criteria were age between 18 and 75 years, Eastern Cooperative Oncology Group (ECOG) performance status 0–1, histologically or cytologically confirmed locally advanced unresectable or metastatic ESCC, progressed after one or more lines of systemic therapy, EGFR overexpression (Immunohistochemistry [IHC] 3 + [staining intensity range 0–3]) or amplification (fluorescence in situ hybridization [FISH] test-positive) detected by the central lab, must have measurable disease based on RECIST 1.1 as determined by the site, and adequate major organ function. The main exclusion criteria were history of other EGFR-targeted therapies, active infection, bleeding, unstable cardiovascular disease, and pregnancy or lactation.

### Procedure

The status of EGFR in tumor specimens was tested in a central lab before enrolment. IHC assays were performed as per the manufacturer’s instructions using CONFIRM anti-EGFR rabbit monoclonal primary antibody (clone 5B7, Roche 790-4347). EGFR IHC staining scores were independently judged by two pathologists according to the evaluation criteria (definition of IHC 3+ = strong cytoplasmic or/and membranous reactivity in ≥ 10% of tumor cells). EGFR amplification was measured using a FISH assay (Vysis EGFR/CEP 7 FISH Probe Kit produced by Abbott, Cat # 01N35-020) counting 50 cells. EGFR-amplified tumors were defined as an average copy number of EGFR ≥ 4, or ≥ 15.0% of all counted cells with EGFR copy number ≥ 6, or an average copy number of less than 4, but EGFR/CEP ratio ≥ 2.0.

Eligible patients received larotinib (Sunshine Lake Pharma Co., Ltd.) orally at 350 mg, 300 mg, or 250 mg daily (28 consecutive days as a treatment cycle) until disease progression, intolerable toxicity, or withdrawal of informed consent. A maximum of two dose reductions was permitted in 50 mg increments due to drug intolerance. Toxicity was assessed according to the National Cancer Institute Common Terminology Criteria for Adverse Events (NCI-CTCAE) Version 4.03. Tumor responses were evaluated by both investigators and independent radiology review every 8 weeks according to the Response Evaluation Criteria in Solid Tumors (RECIST version 1.1). Complete or partial responses were confirmed at a subsequent time point 4 weeks later. The primary efficacy endpoint was investigator-assessed objective response rate (ORR).

### Statistical analysis

At an early phase of the study, the evaluation of efficacy and safety was conducted in 3 dose groups, namely, 250 mg/d, 300 mg/d, and 350 mg/d. After the analysis of certain accumulated data, 350 mg was chosen to expand enrolment to determine its efficacy and safety. We calculated the sample size of the 350 mg group based on the primary efficacy endpoint, which is ORR. According to findings from previous studies, the ORR to second-line therapy was around 5% in ESCC [20, 25], we assumed 5% ORR for the historical control group, and 20% for the larotinib (350 mg) group. Considering a dropout rate of 10%, 45 patients were required to ensure 80% power at a 20% two-tailed significance level.

Subjects who met the selection criteria and received at least one dose of the treatment drug were included in the full analysis set and safety set. The response evaluable set referred to subjects who had a baseline and at least one post-treatment tumor assessment, and were used for the assessment of efficacy endpoints.

For efficacy analysis, the ORR was calculated based on the observable number of cases achieving objective responses (confirmed complete response [CR] plus partial response [PR]). The 95% confidence interval (95% CI) was calculated using the Closure-Pearson method. PFS and OS were calculated using the Kaplan–Meier method and the log-rank test to assess statistical significance. Descriptive statistics and MedDRA medical terminology was used to classify organ systems to summarize the incidence and severity of adverse events and serious adverse events. All analyses were conducted using SAS system version 9.2 (SAS Institute, Cary, NC, USA).

## Results

### Patients

**Fig. 1:**
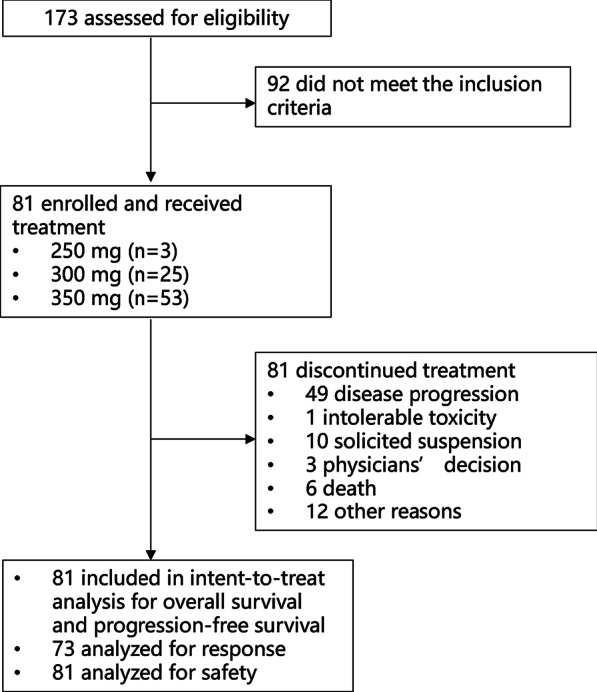
Patient disposition

Between August 8, 2017, and March 01, 2021, a total of 173 patients were screened and 81 eligible subjects were enrolled (Fig. 1). Patients’ baseline characteristics are shown in Table 1. The median age was 61 years (range 38–75), ECOG performance status was 1 in 86.4% of the patients, and the majority (88.9%) had stage IV ESCC. Most (61.7%) patients had received two or more previous lines of systemic therapy. EGFR overexpression was reported in 95.1% of the patients (77/81), EGFR amplification was seen in 26.3% of the patients (20/81), 16 of them had both EGFR overexpression and amplification. The median duration of treatment was 77 days (range 7–789). All subjects had discontinued study treatment, most due to progressive disease (Fig. 1).

**Table 1:** Baseline characteristics

Characteristics	250 mg (n = 3)	300 mg (n = 25)	350 mg (n = 53)	All (n = 81)
*Age*				
Median (range)	55 (52, 58)	63 (47, 71)	59 (38, 75)	61 (38, 75)
< 65	3 (100)	17 (68.0)	41 (77.4)	61 (75.3)
≥ 65	0	8 (32.0)	12 (22.6)	20 (24.7)
*Sex, n (%)*				
Male	2 (66.7)	19 (76)	49 (92.5)	70 (86.4)
Female	1 (33.3)	6 (24)	4 (7.5)	11(13.6)
*Race, n (%)*				
Asian	3 (100)	25 (100)	53 (100)	81 (100)
Others	0	0	0	0
*Height, n (%)*				
Median (range)	170.0 (150, 176)	167.0 (140–178)	170.0 (147, 180)	170.0 (140, 180)
Mean (SD)	165.3 (13.61)	166.1 (8.36)	168.8 (7.17)	167.9 (7.80)
*Weight, n (%)*				
Median (range)	62.00 (42.0, 75.0)	56.00 (42.0, 88.0)	59.00 (40.0, 82.0)	59.00 (40.0, 88.0)
Mean (SD)	59.67 (16.623)	58.95 (10.086)	60.85 (9.649)	60.22 (9.936)
*BMI (kg/m*^*2*^*), n (%)*				
Median (range)	20.00 (18.7, 26.0)	20.70 (16.8, 29.0)	20.80 (14.3, 26.8)	20.80 (14.3, 29.0)
Mean (SD)	21.57 (3.894)	21.32 (2.877)	21.35 (3.179)	21.35 (3.071)
*ECOG, n (%)*				
0	0	2 (8.0)	9 (17.0)	11 (13.6)
1	1 (100)	23 (92.0)	44 (83.0)	70 (86.4)
*TNM classification*				
III			3 ( 5.7)	3 ( 3.7)
IV	3 (100)	25 (100)	47 (88.7)	78 (96.3)
*Metastases, n (%)*				
M_0_	1 (33.3)	0	8 (15.1)	9 (11.1)
M_1_	2 (66.7)	25(100)	45 (84.9)	72 (88.9)
*Prior lines of systemic therapy, n (%)*				
1	2 (66.7)	13 (52.0)	16 (30.2)	31 (38.3)
2	0	7 (28.0)	28 (52.8)	35 (43.2)
≥ 3	1(33.3)	5 (20.0)	9 (17.0)	15 (18.5)
*Prior therapies for ESCC*				
Surgery	1 (33.3)	12 (48.0)	29 (54.7)	42 (51.9)
Radiotherapy	2 (66.7)	18 (72.0)	30 (56.6)	50 (61.7)
Chemotherapy	3 (100)	25 (100)	53 (100)	81 (100)
PD-1/PD-L1 antibodies	0	4 (16.0)	11 (20.8)	15 (18.5)
Target therapy	0	3 (37.5)	5 (25.0)	8 (28.6)
Traditional medicine	0	1 (12.5)	5 (25.0)	6 (21.4)
Other	0	1 (12.5)	0	1 ( 3.6)
*Duration of advanced disease from first diagnosis to informed consent (months)*				
Median (range)	10.3 (3.7, 33.5)	13.2 (4.9, 45.6)	16.0 (3.7, 74.6)	15.3 (3.7, 74.6)
*EGFR IHC staining, n (%)*				
3+	3 (100)	23 (92.0)	51 (96.2)	77 (95.1)
2+	0	2 (8.0)	2 (3.8)	4 (4.9)
*EGFR FISH, n (%)*				
Positive	0	6(24)	14 (29.2)	20 (26.3)
Negative	3 (100)	19(76.0)	34 (70.8)	56 (73.7)

### Responses and survival

**Fig. 2:**
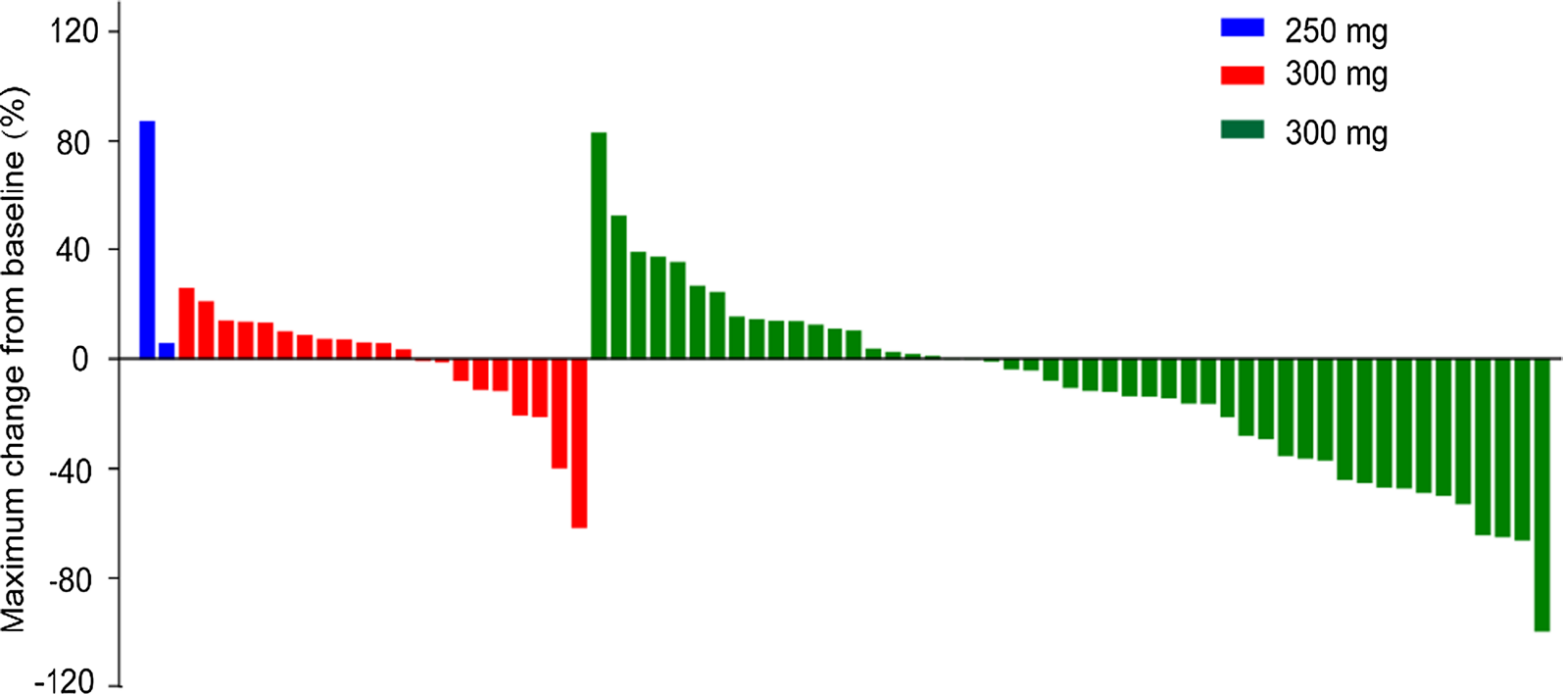
Waterfall plot of best percent change in target lesions from baseline

Seventy-three of 81 patients were evaluated for efficacy by investigators via RECIST v1.1 criteria, and 10 (13.7%) of them had confirmed tumor partial responses (Fig. 2). All tumor responses were observed in the 350 mg group, with 10 of them having EGFR overexpression and 4 having EGFR amplification. Among patients treated with larotinib 350 mg, the ORR was 20.0% (95% CI 10.0–33.7) for all patients and 14.3% (95% CI 4.8–30.3) for patients with 2 or more previous systemic therapies (Table 2). The ORR was 13.9% (10/72; 95% CI 6.9–24.1) assessed by independent radiology review for all patients, and 16.3% (8/50; 95% CI 7.3–29.7) for patients in 350 mg group, with a disease control rate of 61.2% (95% CI 46.2–74.8) (Additional file 3). In all patients, the investigator-assessed median DOR was 6.6 months (95% CI 1.9–12.9) with the longest response being 12.9 months.

**Table 2:** Confirmed overall response by dose levels and by prior lines of therapy

	250 mg	300 mg	350 mg	All
*All patients*				
Number of evaluable patients	n = 2	n = 21	n = 50	n = 73
Complete response, n (%)	0	0	0	0
Partial response, n (%)	0	0	10 (20.0)	10 (13.7)
Stable disease^a^, n (%)	0	12 (57.1)	22 (44.0)	34 (46.6)
Progressive disease, n (%)	2 (100)	7 (33.3)	16 (32.0)	25 (34.2)
Not evaluable, n (%)	0	2 (9.5)	2 (4.0)	4 (5.5)
ORR, %(95% CI)	0 (0–84.2)	0 (0–16.1)	20.0 (10.0, 33.7)	13.7 (6.8, 23.8)
DCR, %(95% CI)	0 (0–84.2)	57.1 (34.0–78.2)	64.0 (49.2–77.1)	60.3 (48.1–71.6)
*Patients with 2 or more prior lines of systemic therapy*				
Number of evaluable patients	n = 0	n = 9	n = 35	n = 44
Complete response, n (%)	NA	0	0	0
Partial response, n (%)	NA	0	5 (14.3)	5 (11.4)
Stable disease^a^, n (%)	NA	4 (44.4)	15 (42.9)	19 (43.2)
Progressive disease, n (%)	NA	4 (44.4)	13 (37.1)	17 (38.6)
Not evaluable, n (%)	NA	1 (11.1)	2(5.7)	3 (6.8)
ORR, % (95% CI)	NA	0 (0.0–33.6)	14.3 (4.8–30.3)	11.4 (3.8–24.6)
DCR, % (95% CI)	NA	44.4 (13.7–78.8)	57.1 (39.4–73.7)	54.5 (38.9–69.6)

^a^Stable disease ≥ 6 weeks

**Fig. 3:**
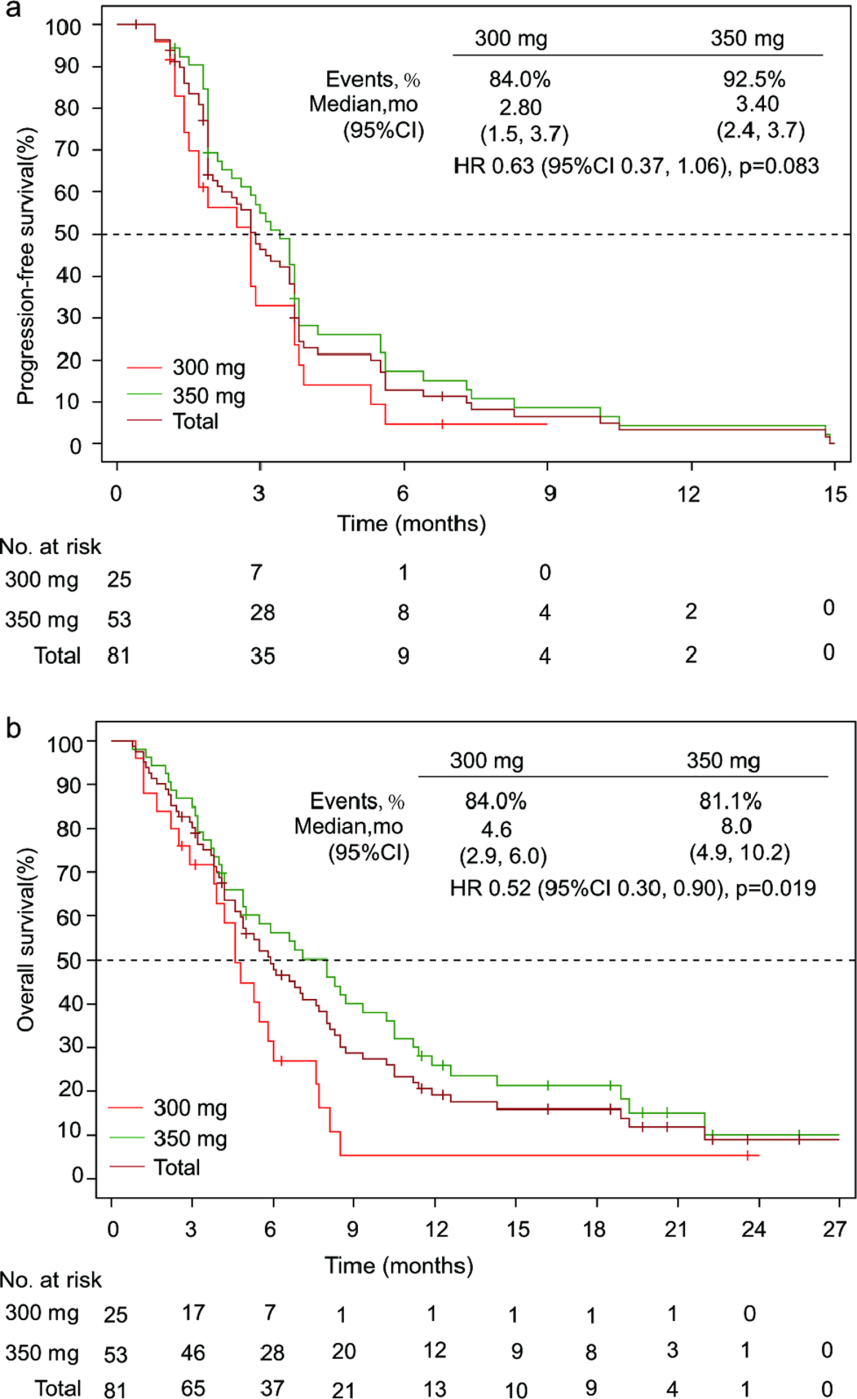
Kaplan–Meier estimates of progression-free survival (**a**) and overall survival (**b**) according to treatment group

As of March 01, 2021, the median duration of follow-up was 20.6 months (IQR 16.2–23.6). The median PFS and OS for all patients were 2.90 (95% CI 2.1–3.6) months and 5.90 (95% CI 4.6–7.7) months, respectively. Longer PFS and OS were both observed in patients treated with loratinib 350 mg, with a median OS 8.0 (95% CI 4.9–10.2) months and PFS 3.4 (95% CI 2.4–3.7) months (Fig. 3). However, those were similar across EGFR overexpression and amplification subgroups (Additional file 4).

### Safety

Of the 81 subjects evaluated for safety, 80 (98.8%) experienced at least one treatment-related adverse event (TRAE). The most common TRAEs were mostly mild (grade 1/2), including diarrhea (67.9%), rash (64.2%), palmar-plantar erythrodysesthesia syndrome (39.5%), oral ulcer (30.9%), and aneamia (30.9%) (Table 3). The most common grade 3 or more TRAEs were rash (8.6%), anemia (6.2%), palmar-plantar erythrodysesthesia syndrome (4.9%), elevated ALT (4.9%), and interstitial lung disease (ILD) or pneumonitis (4.9%). Treatment-related serious adverse events occurred in 14 patients, including ILD or pneumonitis (five), elevated liver enzymes (three), and one each of rash, paronychia, fatigue (grade 2), anorexia (grade 1), hypotension, and sudden death (considered as an unknown relationship with loratinib), TRAEs leading to permanent discontinuation included ILD or pneumonitis (four), and one of each nausea complicated with fatigue, paronychia, sudden death, and rash. Most TRAEs of grade 3 and higher could be recovered or became tolerable after therapeutic intervention. The incidence, type, and severity of larotinib-related TRAEs are consistent with the known safety profile of other first-generation EGFR-TKIs.

**Table 3:** Treatment related adverse events occurring in 10% or more patients

TRAE	250 mg (n = 3)		300 mg (n = 25)		350 mg (n = 53)		Total (n = 81)	
	G-All^a^	≥ G3	G-All	≥ G3	G-All	≥ G3	G-All	≥ G3
*Gastrointestinal disorders (n, %)*								
Diarrhea	3 (100)	0	16 (64.0)	1 (4.0)	36 (67.9)	1 (1.9)	55 (67.9)	2 (2.5)
Oral ulcer	0	0	4 (16.0)	0	21 (39.6)	1 (1.9)	25 (30.9)	1 (1.2)
Vomiting	1 (33.3)	0	8 (32.0)	1 (4.0)	11 (20.8)	0	20 (24.7)	1 (1.2)
Nausea	1 (33.3)	0	8 (32.0)	1 (4.0)	7 (13.2)	1 (1.9)	16 (19.8)	2 (2.5)
Oral mucositis	0	0	3 (12.0)	0	7 (13.2)	0	10 (12.3)	0
*Skin and subcutaneous tissue disorders (n, %)*								
Rash	1 (33.3)	0	10 (40.0)	0	41 (77.4)	7 (13.2)	52 (64.2)	7 (8.6)
Palmar-plantar erythrodysesthesia syndrome	1 (33.3)	1 (33.3)	7 (28.0)	1 (4.0)	24 (45.3)	2 (3.8)	32 (39.5)	4 (4.9)
*Investigations (n, %)*								
Elevated AST	0	0	4 (16.0)	0	17 (32.1)	4 (7.5)	21 (25.9)	4 (4.9)
Elevated ALT	0	0	5 (20.0)	0	15 (28.3)	2 (3.8)	20 (24.7)	2 (2.5)
Leukopenia	0	0	3 (12.0)	0	6 (11.3)	0	9 (11.1)	0
*Metabolism and nutrition disorders (n, %)*								
Anorexia	0	0	2 (8.0)	0	10 (18.9)	1 (1.9)	12 (14.8)	1 (1.2)
Hypoalbuminemia	0	0	0	0	12 (22.6)	0	12 (14.8)	0
*Infections and infestations (n, %)*								
Paronychia	0	0	4 (16.0)	0	7 (13.2)	1 (1.9)	11 (13.6)	1 (1.2)
*General disorders and administration site conditions (n, %)*								
Fatigue	0	0	3 (12.0)	1 (4.0)	9 (17.0)	2 (3.8)	12 (14.8)	3 (3.7)
*Blood and lymphatic system disorders (n, %)*								
Anemia	1 (33.3)	1 (33.3)	4 (16.0)	1 (4.0)	20 (37.7)	3 (5.7)	25 (30.9)	5 (6.2)
*Renal and urinary disorders (n, %)*								
Proteinuria	0	0	3 (12.0)	0	13 (24.5)	0	16 (19.8)	0

^a^G, grade

## Discussion

In this open-label, multi-center study, larotinib demonstrated promising anti-tumor activity and manageable toxicity profiles in previously treated patients with advanced and metastatic ESCC with EGFR overexpression or amplification. Although about 61.7% of patients underwent 2 or more prior therapies, larotinib showed encouraging efficacy, especially at the dose of 350 mg, with a confirmed ORR of 20.0%, median PFS of 3.4 months, and median OS of 8.0 months.

Over the past decades, there were few options for patients with advanced ESCC who have progressed after first-line chemotherapy. Despite systemic chemotherapeutic agents such as docetaxel, paclitaxel, and irinotecan being used for this patient population, clinical benefits were limited. Recently, immune checkpoint inhibitors, such as pembrolizumab, nivolumab, and camrelizumab, showed promising efficacy and were approved as second-line ESCC therapy. These PD-1 antibodies demonstrated better efficacy in pivotal phase 3 studies when compared with chemotherapy in ESCC. The median OS was from 8.2 months to 10.9 months, and ORR ranged from 16.7% to 20.2% [12–14]. Larotinib at 350 mg demonstrated a similar antitumor activity as immune therapy. Even in patients who had undergone 2 or more prior therapies, encouraging antitumor activity was also observed. The ORR was 14.3% at 350 mg, near to that of pembrolizumab (9/63, 14.3%) reported in KEYNOTE-180 [26]. Moreover, 5 of 9 patients who had progressed after immunotherapy had tumor reduction (one had a partial response), suggesting larotinib might represent a new treatment option for patients with ESCC. However, the lack of statistical rigor associated with small sample sizes was problematic, and this study was a single-arm, non-randomized study; these findings should be interpreted with caution because of the different patient compositions in our study.

Even though several studies regarding first-generation EGFR TKIs, including gefitinb, erlotinib, and icotinib, have been conducted in advanced esophageal carcinoma, limited benefits were observed. As mentioned in the introduction section, the main possible reasons behind the failure of gefitinib and erlotinib studies in esophageal carcinoma were: no population screening was conducted, with most of the recruited subjects were diagnosed with adenocarcinoma, or no EGFR biomarker screening was performed. The phase II study of Icotinib in esophageal cancer learned lessons from the gefitinib and erlotinib studies, subjects enrolled were ESCC with EGFR overexpression (an immunohistochemical [IHC] staining score of 3 matriculations) or EGFR gene amplification (a positive fluorescence in situ hybridization [FISH] result), and the ORR increased to 16.7% (9/54); however, median PFS is only 1.7 months, with a median OS of 5.1 months. In this phase Ib clinical study in ESCC, the subject of EGFR biomarker requirement is almost the same as that of icotinib. More importantly, this study demonstrated a higher ORR and improved survival benefit, in the 350 mg group, the median OS is 8.0 months and median PFS is 3.4 months, with an ORR of up to 20.0% (10/50), which may be attributed to the difference in terms of drug property. This could be also explained in preclinical findings (Additional file 5), in which larotinib showed a stronger inhibition activity against EGFR kinase and higher accumulation in esophageal tissue than icotinib did. Moreover, in comparison with its counterparts, such as gefitinib and erlotinib, larotinib exhibited a similar effect on EGFR kinase but higher accumulation in esophageal tissue (Additional file 1). Besides, larotinib also demonstrated a higher ratio of exposure in tumor/plasma when compared with erlotinib (Additional file 6).

Even though both EGFR overexpression and amplification were selected to be predictors of efficacy in our study, similar survival benefits were observed between patients from two subgroups, with the median OS of both 5.9 months and PFS of 3.6 months versus 3.8 months. The reason was probably that almost all patients with EGFR amplification had EGFR overexpression (only 4 patients showed EGFR amplification but low expression), suggesting that EGFR overexpression might be correlated with EGFR amplification, which corroborated previous findings [16, 23]. Considering 95% of patients in our study were EGFR-overexpressed, and 10 responses all occurred in this patient population, EGFR overexpression could be used as a predicted biomarker in the future study of larotinib in ESCC.

Larotinib is well-tolerated in patients with ESCC. The toxicity profile in our study was similar to that of other first-generation EGFR-TKIs. Most of the TRAEs were grades 1 or 2, and most of the treatment-related SAEs could be resolved or became tolerable after therapeutic interventions. Although interstitial lung disease (ILD) was observed (five patients) in our study. Among them, two were recently pre-treated with anti-PD-1 monoclonal antibodies. The time intervals from the last dose of PD-1 inhibitor and the first dose of larotinib in the two subjects were 33 days and 34 days, respectively. Interestingly, no ILD was reported in the patients with intervals longer than 50-days, suggesting that the time interval between anti-PD-1 antibody administration and larotinib treatment may be related to the occurrence of ILD. A similar finding was observed in patients with NSCLC treated with sequential anti-PD-(L)1 monoclonal antibody followed by osimertinib [27, 28]. The washout period of immunotherapy, anti-PD-(L)1 monoclonal antibody, in particular, should be long enough, at least 50-days, in follow-up studies of larotinib to minimize inadvertent but possible serious toxicity.

Despite the encouraging results in this study, there remain several limitations. The study outcomes might be biased by the small sample size and the single-arm design. Furthermore, quality of life was not assessed in this study. Thus, a randomized, open-label, phase 3 clinical trial of larotinib in over 400 patients is ongoing (ClinicalTrials.gov identifier NCT04415853).

## Conclusions

In conclusion, larotinib showed encouraging antitumor activity and a tolerable safety profile in pre-treated advanced ESCC patients with EGFR overexpression or amplification, especially at the dose of 350 mg. A phase 3 study is underway to further evaluate the efficacy and safety of 350 mg larotinib for the treatment of ESCC with EGFR overexpression (ClinicalTrials.gov identifier NCT04415853).

## Supplementary Information

**Additional file 1**. Which is entitled with Preclinical studies on Xenografted Mice Models, is an in vivo assay reporting results regarding the evaluation of antitumor efficacy of larotinib in xenografted mice models, plasma, and tumor tissues.

**Additional file 2**. Which is entitled with Supplementary Materials and Methods, is a description about protocol revision history for study PCD-DZ650-17-001 (NCT03888092).

**Additional file 3**. Which is entitled with Overall Response Assessed by Independent Radiology Review, is a supplementary table describing confirmed overall response assessed by independent radiology review.

**Additional file 4**. Which is entitled with Progression-free Survival and Overall Survival in Subgroups, is a supplementary table describing subgroup analyses of Progression-free Survival and Overall Survival at different dose levels.

**Additional file 5**. Which is entitled with Preclinical Kinase Inhibition Assays, is a report describing a preclinical study designed to make comparison of inhibitory activity on EGFR family kinases in the same batch among different first-generation EGFR-TKIs.

**Additional file 6**. Which is entitled with Preclinical Tissue Distribution Study, describes a preclinical tumor distribution study designed to confirm whether larotinib had a better distribution than other EGFR-TKIs in esophageal tumor tissues.

**Additional file 7**. Which is entitled with Baseline characteristics, is Table 1 that is cited and indicated within the submitted manuscript. Since this table is larger than one A4, it is uploaded as an additional file.

**Additional file 8**. Which is entitled with Treatment related adverse events occurring in 10% or more patients, is Table 3 that is cited and indicated within the submitted manuscipt. Since this table is larger than one A4, it is uploaded as an additional file.

## Data Availability

The datasets used and/or analyzed during the current study are available from the corresponding author on reasonable request.
