# Supplementary material for: Larotinib in patients with advanced and previously treated esophageal squamous cell carcinoma with epidermal growth factor receptor overexpression or amplification: an open-label, multicenter phase 1b study
Source: BMC Gastroenterol. 2021 Oct 23;21:398. doi: 10.1186/s12876-021-01982-4 (PMC8540164; doi:10.1186/s12876-021-01982-4)
Supplement: Supplementary file 1 — Additional file 1. Which is entitled with Preclinical studies on Xenografted Mice Models, is an in vivo assay reporting results regarding the evaluation of antitumor efficacy of larotinib in xenografted mice models, plasma, and tumor tissues. [file 12876_2021_1982_MOESM1_ESM.pdf]

Additional file

Article title: Larotinib in Patients with Advanced and Previously Treated Esophageal Squamous Cell Carcinoma with Epidermal Growth Factor Receptor Overexpression or Amplification: An Open-Label, Multicenter Phase 1b Study

Journal name: Cancer Chemotherapy and Pharmacology

Author names: Jianming Xu, Lianke Liu, Rongrui Liu, Chuanhua Zhao, Yuxian Bai, Yulong Zheng, Shu Zhang, Ning Li, Jianwei Yang, Qingxia Fan, Xiuwen Wang, Shan Zeng, Yingjun Zhang, Weihong Zhang, Yulei Zhuang, Ning Kang, Yingzhi Jiang, Hongmei Sun

Lianke Liu, Rongrui Liu and Chuanhua Zhao contributed equally to this work, and are considered as joint first authors.

Corresponding authors: Jianming Xu, jmxu2003@yahoo.com

Additional file 1: Preclinical studies on Xenografted Mice Models

Purpose: This *in vivo* assay aimed to evaluate antitumor efficacy of larotinib in xenografted mice models, plasma, and tumor tissues.

Methods: Female BALB/cA−nude mice (5–6 weeks old) were utilized in this study. Human tumor xenografts were established by s.c. inoculation of BxPC-3 cells in nude mice. Tumour-bearing mice were randomized into 5 groups based on tumour size, including 4 treatment groups (24 animals per treatment group) and 1 vehicle control group (10 animals in vehicle control group). Larotinib and erlotinib were administered once daily by oral gavage for a treatment duration of 18 days with 18, 36, 72 mg/kg doses for larotinib (equal to the free alkali doses of 12.5, 25 50 mg/kg), and with 55 mg /kg for erlotinib (equal to the free alkali dose of 50 mg/kg), when average tumor volume reached approximately 150 mm^3. The measurement of subcutaneous tumor size and body weight was performed twice a week. Tumor volume was determined by measuring two perpendicular diameters using callipers, and was calculated as $(\text{length} \times \text{width}^2)/2$. The data were expressed as means ± SEM and were plotted as a function of time. Statistical analyses were conducted using one-way ANOVA with p value set at 0.05, including drug-treated tumors versus vehicle-treated tumors and drug-treated tumors versus pre-dosed tumors. Each group received oral administration again after 18-day treatment. Blood and tumor tissues were collected subsequently at 0, 0.5, 1, 2, 4, 8, 12, and 24 h. Plasma was separated and stored at -80 °C, and tumor tissues were cryopreserved in liquid nitrogen. Larotinib and erlotinib concentrations in plasma and tumor homogenate were measured subsequently.

Results: This assay reported significant tumor growth inhibition when larotinib was administered in BxPC3 xenograft models, and it was shown that the concentrations were high in tumor tissues.

Larotinib demonstrated significant anti-tumor activity in all *in vivo* studies. In the BxPC3 model, when compared with vehicle control group, larotinib greatly inhibited tumor growth, and exhibited tumor growth inhibition (%TGI) values of 79% ($P<0.001$), 96% ($P<0.001$), and 109% ($P<0.001$) at doses of 18, 36, and 72 mg/kg, respectively. The dose of 55 mg/kg erlotinib demonstrated significant tumor inhibition effect with %TGI value of 94% ($P<0.001$) (Fig. S1).

We chose to examine larotinib and erlotinib concentrations in tumors and plasma from mice bearing BxPC3 xenografts. Larotinib and erlotinib concentrations versus time profiles in blood and tumor are shown in Figure S2 and Table S1. The tumor inhibition rate of larotinib (18 mg/kg, PO, QD 18) in the subcutaneous transplantation tumor of BXPC-3 nude mice was 79%. The drug concentrations in plasma and tumor tissues at 24 h after the last administration were about 4 ng/ml and 2452 ng/g, respectively. The drug exposure levels in plasma and tumor tissues (AUC) were 3364.5 h*ng/ml and 92442.8 h*ng/g, respectively. We speculated to establish the relatively effective tumor inhibition effect after continuous administration of larotinib dimesylate (such as tumor inhibition rate of 79%). Drug efficacy could be sustained by maintaining drug concentration in plasma and tumor tissues above 4 ng/ml and 2450 ng/g, respectively, and drug exposure above 3360 h*ng/ml and 92440 h*ng/g, respectively, 24 h after administration. Compared to erlotinib, a higher ratio of drug concentration was observed in tumor tissue/plasma for larotinib, suggesting significant improvement of anti-tumor activity against other EGFR TKIs.

Table S1 Pharmacokinetic parameters and tumor inhibition rate in plasma and tumor tissues of BxPC-3 xenograft model

**Parameters**	**Larotinib**						**Erlotinib**	
	18 mg/kg		36 mg/kg		72 mg/kg		55 mg/kg	
	Plasma	Tumors	Plasma	Tumors	Plasma	Tumors	Plasma	Tumors
**AUC_{INF}**	3402.6	329554.2	8323.1	875964.1	18458.2	/	56001.8	36900.7
**AUC_{last}**	3364.5	92442.8	8263.4	263064.0	18369.9	452938.8	52331.1	34547.2
**C_{max}**	651.6	5433.4	1290.7	12545.3	2473.3	26179.7	16599.0	10003.1
**MRT_{INF}(h)**	6.0	72.2	6.0	66.8	6.5	/	5.6	5.6
**$T_{1/2}$(h)**	3.8	49.9	3.5	46.1	2.8	/	4.1	4.0
**T_{max}(h)**	1	4	1	4	1	8.0	0.5	0.5
**TGI(%)**	79		96		109		94	

Unit: AUC, h*ng/ml for plasma and h*ng/g for tumor tissues; C_{max}, ng/ml for plasma and ng/g for tumor tissues

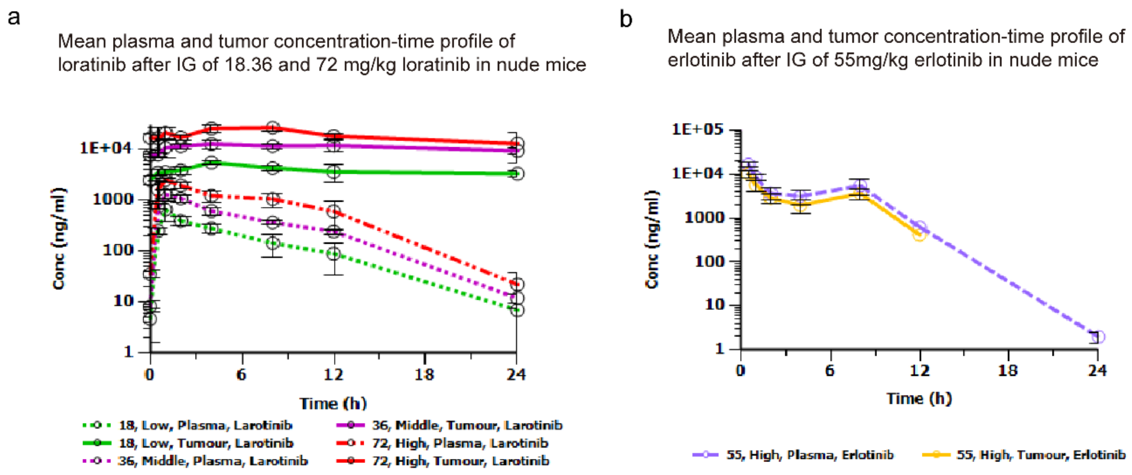

Fig. S1 Concentration of larotinib (a) and erlotinib (b) in tumor tissues and plasma
