# Supplementary material for: Larotinib in patients with advanced and previously treated esophageal squamous cell carcinoma with epidermal growth factor receptor overexpression or amplification: an open-label, multicenter phase 1b study
Source: BMC Gastroenterol. 2021 Oct 23;21:398. doi: 10.1186/s12876-021-01982-4 (PMC8540164; doi:10.1186/s12876-021-01982-4)
Supplement: Supplementary file 2 — Additional file 2. Which is entitled with Supplementary Materials and Methods, is a description about protocol revision history for study PCD-DZ650-17-001 (NCT03888092). [file 12876_2021_1982_MOESM2_ESM.pdf]

Additional file 2

Article title: Larotinib in Patients with Advanced and Previously Treated Esophageal Squamous Cell Carcinoma with Epidermal Growth Factor Receptor Overexpression or Amplification: An Open-Label, Multicenter Phase 1b Study

Journal name: Cancer Chemotherapy and Pharmacology

Author names: Jianming Xu, Lianke Liu, Rongrui Liu, Chuanhua Zhao, Yuxian Bai, Yulong Zheng, Shu Zhang, Ning Li, Jianwei Yang, Qingxia Fan, Xiuwen Wang, Shan Zeng, Yingjun Zhang, Weihong Zhang, Yulei Zhuang, Ning Kang, Yingzhi Jiang, Hongmei Sun

Lianke Liu, Rongrui Liu and Chuanhua Zhao contributed equally to this work, and are considered as joint first authors.

Corresponding authors: Jianming Xu, jmxu2003@yahoo.com

Additional file 2: Supplementary Materials and Methods

Protocol revisions

The study was originally designed as a randomized, open-label, multi-center clinical trial, the treatment groups were 350 mg and 300 mg. After enrolling 10 patients, there were 4 patients (3 of 5 patients in the 350 mg group and 1 of 5 patients in the 300 mg group) required dose reduction due to intolerability. A protocol amendment was made with the dose groups adjusted to 300 mg and 250 mg to ensure subject safety. However, when more safety and efficacy data were collected (one patient in 250 mg group, 15 patients in each 300 mg and 350 mg group), we re-evaluated the safety and efficacy of these three doses, and determined 350 mg to be the effective dose. Therefore, the study was ultimately designed as a single-arm, open-label study at 350 mg dose.
