# Supplementary material for: Larotinib in patients with advanced and previously treated esophageal squamous cell carcinoma with epidermal growth factor receptor overexpression or amplification: an open-label, multicenter phase 1b study
Source: BMC Gastroenterol. 2021 Oct 23;21:398. doi: 10.1186/s12876-021-01982-4 (PMC8540164; doi:10.1186/s12876-021-01982-4)
Supplement: Supplementary file 3 — Additional file 3. Which is entitled with Overall Response Assessed by Independent Radiology Review, is a supplementary table describing confirmed overall response assessed by independent radiology review. [file 12876_2021_1982_MOESM3_ESM.pdf]

Additional file

Article title: Larotinib in Patients with Advanced and Previously Treated Esophageal Squamous Cell Carcinoma with Epidermal Growth Factor Receptor Overexpression or Amplification: An Open-Label, Multicenter Phase 1b Study

Journal name: Cancer Chemotherapy and Pharmacology

Author names: Jianming Xu, Lianke Liu, Rongrui Liu, Chuanhua Zhao, Yuxian Bai, Yulong Zheng, Shu Zhang, Ning Li, Jianwei Yang, Qingxia Fan, Xiuwen Wang, Shan Zeng, Yingjun Zhang, Weihong Zhang, Yulei Zhuang, Ning Kang, Yingzhi Jiang, Hongmei Sun

Lianke Liu, Rongrui Liu and Chuanhua Zhao contributed equally to this work, and are considered as joint first authors.

Corresponding authors: Jianming Xu, jmxu2003@yahoo.com

Additional file 3: Overall Response Assessed by Independent Radiology Review

Table S2 Confirmed overall response assessed by independent radiology review

All patients	250 mg	300 mg	350 mg	All
	**n=2**	**n=21**	**n=49[a]**	**n=72[a]**
CR, n(%)	0	0	0	0
PR, n(%)	0	2 (9.5)	8 (16.3)	10 (13.9)
SD, n(%)	0	8 (38.1)	22 (44.9)	30 (41.7)
PD, n(%)	2 (100)	9 (42.9)	15 (30.6)	26 (36.1)
NE, n(%)	0	2 (9.5)	4 (8.2)	6 (8.3)
**ORR, %(95% CI)**	0 (0.0, 84.2)	9.5 (1.2, 30.4)	16.3(7.3, 29.7)	13.9 (6.9, 24.1)
**DCR , %(95% CI)**	0 (0.0, 84.2)	47.6 (25.7, 70.2)	61.2 (46.2, 74.8)	55.6 (43.4, 67.3)

Abbreviations: CR, complete response; PR, partial response; SD, stable disease ⩾ 6 weeks; PD, progressive disease; NE, not evaluable; ORR, objective response rate; DCR, disease control rate, including CR, PR and SD; CI, confidence interval.

[a] The image of one patient in 350 mg group was evaluated by investigator but not by independent radiology review due to image quality.
