# Supplementary material for: Larotinib in patients with advanced and previously treated esophageal squamous cell carcinoma with epidermal growth factor receptor overexpression or amplification: an open-label, multicenter phase 1b study
Source: BMC Gastroenterol. 2021 Oct 23;21:398. doi: 10.1186/s12876-021-01982-4 (PMC8540164; doi:10.1186/s12876-021-01982-4)
Supplement: Supplementary file 4 — Additional file 4. Which is entitled with Progression-free Survival and Overall Survival in Subgroups, is a supplementary table describing subgroup analyses of Progression-free Survival and Overall Survival at different dose levels. [file 12876_2021_1982_MOESM4_ESM.pdf]

**Additional file**

**Article title:** Larotinib in Patients with Advanced and Previously Treated Esophageal Squamous Cell Carcinoma with Epidermal Growth Factor Receptor Overexpression or Amplification: An Open-Label, Multicenter Phase 1b Study

**Journal name:** Cancer Chemotherapy and Pharmacology

**Author names:** Jianming Xu, Lianke Liu, Rongrui Liu, Chuanhua Zhao, Yuxian Bai, Yulong Zheng, Shu Zhang, Ning Li, Jianwei Yang, Qingxia Fan, Xiuwen Wang, Shan Zeng, Yingjun Zhang, Weihong Zhang, Yulei Zhuang, Ning Kang, Yingzhi Jiang, Hongmei Sun

Lianke Liu, Rongrui Liu and Chuanhua Zhao contributed equally to this work, and are considered as joint first authors.

**Corresponding authors:** Jianming Xu, jmxu2003@yahoo.com

## Additional file 4: Progression-free Survival and Overall Survival in Subgroups

**Table S3 Progression-free survival and overall survival in subgroups**

| | 250 mg | 300 mg | 350 mg | All |
|---|---|---|---|---|
| **All patients** | **n=3** | **n=25** | **n=53** | **n=81** |
| PFS, median (95% CI) | 1.90 (1.40, -) | 2.80 (1.50, 3.70) | 3.40 (2.40, 3.70) | 2.90 (2.10, 3.60) |
| OS, median (95% CI) | 6.10 (1.40, -) | 4.60 (2.90, 6.00) | 8.00 (4.90, 10.20) | 5.90 (4.60, 7.70) |
| **≥2 prior lines of therapy** | **n=1** | **n=12** | **n=37** | **n=50** |
| PFS, median (95% CI) | 1.40 | 1.50 (1.10, 3.70) | 3.40 (1.90, 3.70) | 2.80 (1.90, 3.70) |
| OS, median (95% CI) | 1.40 | 5.50 (1.20, 8.10) | 7.10 (4.20, 10.50) | 6.80 (4.60, 8.50) |
| **EGFR IHC staining 3+** | **n=3** | **n=23** | **n=51** | **n=77** |
| PFS, median (95% CI) | 1.90 (1.40, -) | 2.50 (1.40, 3.70) | 3.60 (2.60, 3.70) | 2.90 (2.10, 3.60) |
| OS, median (95% CI) | 6.10 (1.40, -) | 4.60 (2.90, 5.80) | 8.00 (4.90, 10.50) | 5.90 (4.60, 7.70) |
| **EGFR IHC staining 2+** | **n=0** | **n=2** | **n=2** | **n=4** |
| PFS, median (95% CI) | NA | 3.35 (2.80, -) | 1.90 (-, -) | 2.35 (1.90, -) |
| OS, median (95% CI) | NA | 6.20 (3.90, -) | 5.85 (3.20, -) | 6.20 (3.20, -) |
| **EGFR FISH positive** | **n=0** | **n=6** | **n=14** | **n=20** |
| PFS, median (95% CI) | NA | 2.80 (1.20, -) | 3.75 (1.90, 5.60) | 3.70 (1.90, 5.50) |
| OS, median (95% CI) | NA | 4.05 (1.20, -) | 7.70 (3.70, 12.60) | 5.85 (3.80, 8.50) |
| **EGFR FISH negative** | **n=0** | **n=6** | **n=14** | **n=20** |
| PFS, median (95% CI) | NA | 2.80 (1.20, -) | 3.75 (1.90, 5.60) | 3.70 (1.90, 5.50) |
| OS, median (95% CI) | NA | 4.05 (1.20, -) | 7.70 (3.70, 12.60) | 5.85 (3.80, 8.50) |

Abbreviations: PFS, progression-free survival; OS, overall survival; CI, confidence interval; EGFR,

epidermal growth factor receptor; IHC, Immunohistochemistry; FISH, fluorescence in situ hybridization; NA, not available.
