# Supplementary material for: Larotinib in patients with advanced and previously treated esophageal squamous cell carcinoma with epidermal growth factor receptor overexpression or amplification: an open-label, multicenter phase 1b study
Source: BMC Gastroenterol. 2021 Oct 23;21:398. doi: 10.1186/s12876-021-01982-4 (PMC8540164; doi:10.1186/s12876-021-01982-4)
Supplement: Supplementary file 5 — Additional file 5. Which is entitled with Preclinical Kinase Inhibition Assays, is a report describing a preclinical study designed to make comparison of inhibitory activity on EGFR family kinases in the same batch among different first-generation EGFR-TKIs. [file 12876_2021_1982_MOESM5_ESM.pdf]

Additional file

Article title: Larotinib in Patients with Advanced and Previously Treated Esophageal Squamous Cell Carcinoma with Epidermal Growth Factor Receptor Overexpression or Amplification: An Open-Label, Multicenter Phase 1b Study

Journal name: Cancer Chemotherapy and Pharmacology

Author names: Jianming Xu, Lianke Liu, Rongrui Liu, Chuanhua Zhao, Yuxian Bai, Yulong Zheng, Shu Zhang, Ning Li, Jianwei Yang, Qingxia Fan, Xiuwen Wang, Shan Zeng, Yingjun Zhang, Weihong Zhang, Yulei Zhuang, Ning Kang, Yingzhi Jiang, Hongmei Sun

Lianke Liu, Rongrui Liu and Chuanhua Zhao contributed equally to this work, and are considered as joint first authors.

Corresponding authors: Jianming Xu, jmxu2003@yahoo.com

Additional file 5: Preclinical Kinase Inhibition Assays

Purpose: To understand the difference in activity between larotinib and other similar drugs, we compared the inhibitory activity of larotinib, gefitinib, erlotinib and icotinib on EGFR family kinases in the same batch.

Methods: The inhibition profile of larotinib and other similar drugs against EGFR family kinases was determined using Z′-LYTE biochemical assay and the LanthaScreen Eu Kinase Binding Assay. To determine half-maximal inhibitory concentration (IC50) values, we set ATP concentrations at the Km for each kinase and measured phosphorylation of peptide substrate polymers.

Results: To assess the potential of larotinib to inhibit EGFR family kinases, and to compare its activity with that of the other EGFR inhibitors, we screened the compounds against 11 EGFR family kinases. The results showed that larotinib inhibited the kinase activity of wild-type EGFR and L858R-mutant EGFR significantly, and had potent binding affinity for del19–EGFR. The activities of gefitinib and erlotinib against EGFR-wt and EGFR-mut were similar to those of larotinib. However, larotinib had a stronger ability to inhibit EGFR kinase compared to icotinib (Table S4).

Table S4 *In vitro* inhibitory activity against EGFR-family kinases of larotinib and similar drugs

Kinase	IC_{50} (nM)			
	Larotinib	Gefitinib	Erlotinib	Icotinib
	Z650	Z490	Z621	Z773
EGFR (ErbB1) C797S	7.24	7.18	11.7	38.7
EGFR (ErbB1) G719C	3.54	8.64	3.29	13.1
EGFR (ErbB1) G719S	7.05	10.1	5.84	15.7
EGFR (ErbB1) L858R	0.254	0.452	0.467	2.03
EGFR (ErbB1) L861Q	0.298	0.369	0.269	1.25
EGFR (ErbB1) T790M C797S L858R	264	402	406	1390
EGFR (ErbB1) T790M L858R	101	168	231	1140
EGFR (ErbB1) T790M	40.9	161	368	1080
EGFR (ErbB1)	0.371	0.374	0.554	1.54
EGFR (ErbB1) d746-750	0.203	<0.150	0.248	1.04
EGFR (ErbB1) d747-749 A750P	0.330	0.277	0.342	1.30
