# Supplementary material for: Larotinib in patients with advanced and previously treated esophageal squamous cell carcinoma with epidermal growth factor receptor overexpression or amplification: an open-label, multicenter phase 1b study
Source: BMC Gastroenterol. 2021 Oct 23;21:398. doi: 10.1186/s12876-021-01982-4 (PMC8540164; doi:10.1186/s12876-021-01982-4)
Supplement: Supplementary file 6 — Additional file 6. Which is entitled with Preclinical Tissue Distribution Study, describes a preclinical tumor distribution study designed to confirm whether larotinib had a better distribution than other EGFR-TKIs in esophageal tumor tissues. [file 12876_2021_1982_MOESM6_ESM.pdf]

Additional file

Article title: Larotinib in Patients with Advanced and Previously Treated Esophageal Squamous Cell Carcinoma with Epidermal Growth Factor Receptor Overexpression or Amplification: An Open-Label, Multicenter Phase 1b Study

Journal name: Cancer Chemotherapy and Pharmacology

Author names: Jianming Xu, Lianke Liu, Rongrui Liu, Chuanhua Zhao, Yuxian Bai, Yulong Zheng, Shu Zhang, Ning Li, Jianwei Yang, Qingxia Fan, Xiuwen Wang, Shan Zeng, Yingjun Zhang, Weihong Zhang, Yulei Zhuang, Ning Kang, Yingzhi Jiang, Hongmei Sun

Lianke Liu, Rongrui Liu and Chuanhua Zhao contributed equally to this work, and are considered as joint first authors.

Corresponding authors: Jianming Xu, jmxu2003@yahoo.com

Additional file 6: Preclinical Tissue Distribution Study

Purpose: This tumor distribution study was aimed to confirm whether larotinib had a better distribution than other EGFR-TKIs in esophageal tumor tissues.

Methods: SD rats (males) were given as single daily dose of 5 mg/kg larotinib as well as other EGFR-TKIs (oral administration). Subsequently, the rats were anesthetized at 1, 4, and 8 h. Blood was collected via caudal vein and transferred into heparinized tubes immediately. The collected samples were centrifuged at 3000 rpm for 15 min, and the supernatant plasma was transferred into another tube and stored at −70 °C until analysis. Stomach, lung, liver, esophagus, colon, pancreas, rectum, galactophore and prostate were excised, rinsed with 0.9% (weight volume) saline, and gently blotted with absorbent paper to remove surface saline and blood. The obtained samples were labelled and stored at -70 °C until analysis.

Results: The mean concentration-time profiles of each tissue are summarized in Figure S2, indicating that larotinib was well absorbed after oral administration. This was attributed to the fact that all the tissue concentrations were kept at higher levels after administration at 1, 4 and 8 h. We also found that larotinib showed higher concentrations than other EGFR-TKIs, especially in esophageal tissues. The ratio of esophagus tissue/plasma was 61 times that of EGFR-TKI at 1 h after treatment (Fig. S2; Table S5).

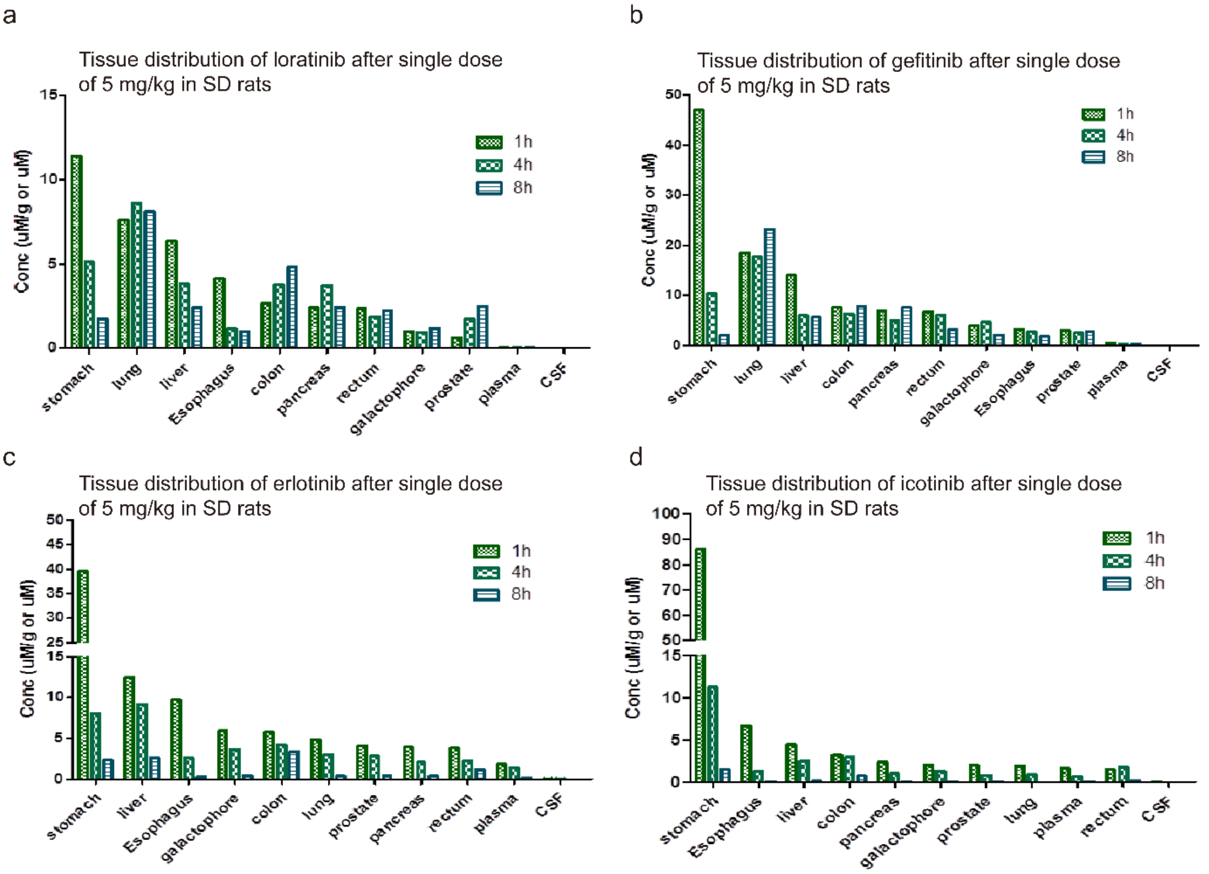

Fig. S2 The mean concentration-time profile of each tissue sample. a) larotinib; b) gefitinib; c) erlotinib; d) icotinib

Table S5 Ratio of esophageal /plasma of different EGFR-TKIs at different time points

Drugs/Time	Ratio of esophageal /plasma		
	1h	4h	8h
Larotinib	61	35	17
Gefitinib	7.6	13	5.5
Erlotinib	5.2	2	1.5
Icotinib	4.1	1.9	1.9
