# Supplementary material for: Larotinib in patients with advanced and previously treated esophageal squamous cell carcinoma with epidermal growth factor receptor overexpression or amplification: an open-label, multicenter phase 1b study
Source: BMC Gastroenterol. 2021 Oct 23;21:398. doi: 10.1186/s12876-021-01982-4 (PMC8540164; doi:10.1186/s12876-021-01982-4)
Supplement: Supplementary file 7 — Additional file 7. Which is entitled with Baseline characteristics, is Table 1 that is cited and indicated within the submitted manuscript. Since this table is larger than one A4, it is uploaded as an additional file. [file 12876_2021_1982_MOESM7_ESM.pdf]

Additional file

Article title: Larotinib in Patients with Advanced and Previously Treated Esophageal Squamous Cell Carcinoma with Epidermal Growth Factor Receptor Overexpression or Amplification: An Open-Label, Multicenter Phase 1b Study

Journal name: Cancer Chemotherapy and Pharmacology

Author names: Jianming Xu, Lianke Liu, Rongrui Liu, Chuanhua Zhao, Yuxian Bai, Yulong Zheng, Shu Zhang, Ning Li, Jianwei Yang, Qingxia Fan, Xiuwen Wang, Shan Zeng, Yingjun Zhang, Weihong Zhang, Yulei Zhuang, Ning Kang, Yingzhi Jiang, Hongmei Sun

Lianke Liu, Rongrui Liu and Chuanhua Zhao contributed equally to this work, and are considered as joint first authors.

Corresponding authors: Jianming Xu, jmxu2003@yahoo.com

Additional file 7: Baseline characteristics

Table 1 Baseline characteristics

Characteristics	250 mg (n=3)	300 mg (n=25)	350 mg (n=53)	ALL (n=81)
**Age**				
**Median(range)**	55 (52,58)	63 (47,71)	59 (38,75)	61 (38, 75)
**<65**	3 (100)	17 (68.0)	41 (77.4)	61 (75.3)
**≥65**	0	8 (32.0)	12 (22.6)	20 (24.7)
**Sex, n (%)**				
**Male**	2 (66.7)	19 (76)	49 (92.5)	70 (86.4)
**Female**	1 (33.3)	6 (24)	4 (7.5)	11(13.6)
**Race, n (%)**				
**Asian**	3 (100)	25 (100)	53 (100)	81 (100)
**Others**	0	0	0	0
**Height, n (%)**				
**Median (range)**	170.0 (150,176)	167.0 (140 - 178)	170.0 (147, 180)	170.0 (140, 180)
**Mean (SD)**	165.3 (13.61)	166.1 (8.36)	168.8 (7.17)	167.9 (7.80)
**Weight, n (%)**				
**Median (range)**	62.00 (42.0, 75.0)	56.00 (42.0, 88.0)	59.00 (40.0, 82.0)	59.00 (40.0, 88.0)
**Mean (SD)**	59.67 (16.623)	58.95 (10.086)	60.85 (9.649)	60.22 (9.936)
**BMI (kg/m^2) , n (%)**				
**Median (range)**	20.00 (18.7, 26.0)	20.70 (16.8, 29.0)	20.80 (14.3, 26.8)	20.80 (14.3, 29.0)
**Mean (SD)**	21.57 (3.894)	21.32 (2.877)	21.35 (3.179)	21.35 (3.071)
**ECOG, n (%)**				
**0**	0	2 (8.0)	9 (17.0)	11 (13.6)
**1**	1 (100)	23 (92.0)	44 (83.0)	70 (86.4)
**TNM classification**				
**III**			3 (5.7)	3 (3.7)
**IV**	3 (100)	25 (100)	47 (88.7)	78 (96.3)
**Metastases, n (%)**				
**M_0**	1 (33.3)	0	8 (15.1)	9 (11.1)
**M_1**	2 (66.7)	25(100)	45 (84.9)	72 (88.9)
**Prior lines of systemic therapy, n (%)**				
**1**	2 (66.7)	13 (52.0)	16 (30.2)	31 (38.3)
**2**	0	7 (28.0)	28 (52.8)	35 (43.2)
**≥3**	1(33.3)	5 (20.0)	9 (17.0)	15 (18.5)
**Prior therapies for ESCC**				

**Surgery**	1 (33.3)	12 (48.0)	29 (54.7)	42 (51.9)
**Radiotherapy**	2 (66.7)	18 (72.0)	30 (56.6)	50 (61.7)
**Chemotherapy**	3 (100)	25 (100)	53 (100)	81 (100)
**PD-1/PD-L1 antibodies**	0	4 (16.0)	11 (20.8)	15 (18.5)
**Target therapy**	0	3 (37.5)	5 (25.0)	8 (28.6)
**Traditional medicine**	0	1 (12.5)	5 (25.0)	6 (21.4)
**Other**	0	1 (12.5)	0	1 (3.6)
**Duration of advanced disease from first diagnosis to informed consent (months)**				
**Median(range)**	10.3 (3.7,33.5)	13.2 (4.9,45.6)	16.0 (3.7,74.6)	15.3 (3.7,74.6)
**EGFR IHC staining, n (%)**				
**3+**	3 (100)	23 (92.0)	51 (96.2)	77 (95.1)
**2+**	0	2 (8.0)	2 (3.8)	4 (4.9)
**EGFR FISH, n (%)**				
**Positive**	0	6(24)	14 (29.2)	20 (26.3)
**Negative**	3 (100)	19(76.0)	34 (70.8)	56 (73.7)
