# Supplementary material for: Larotinib in patients with advanced and previously treated esophageal squamous cell carcinoma with epidermal growth factor receptor overexpression or amplification: an open-label, multicenter phase 1b study
Source: BMC Gastroenterol. 2021 Oct 23;21:398. doi: 10.1186/s12876-021-01982-4 (PMC8540164; doi:10.1186/s12876-021-01982-4)
Supplement: Supplementary file 8 — Additional file 8. Which is entitled with Treatment related adverse events occurring in 10% or more patients, is Table 3 that is cited and indicated within the submitted manuscipt. Since this table is larger than one A4, it is uploaded as an additional file. [file 12876_2021_1982_MOESM8_ESM.pdf]

Additional file

Article title: Larotinib in Patients with Advanced and Previously Treated Esophageal Squamous Cell Carcinoma with Epidermal Growth Factor Receptor Overexpression or Amplification: An Open-Label, Multicenter Phase 1b Study

Journal name: Cancer Chemotherapy and Pharmacology

Author names: Jianming Xu, Lianke Liu, Rongrui Liu, Chuanhua Zhao, Yuxian Bai, Yulong Zheng, Shu Zhang, Ning Li, Jianwei Yang, Qingxia Fan, Xiuwen Wang, Shan Zeng, Yingjun Zhang, Weihong Zhang, Yulei Zhuang, Ning Kang, Yingzhi Jiang, Hongmei Sun

Lianke Liu, Rongrui Liu and Chuanhua Zhao contributed equally to this work, and are considered as joint first authors.

Corresponding authors: Jianming Xu, jmxu2003@yahoo.com

Additional file 8: Treatment related adverse events occurring in 10% or more patients

Table 3 Treatment related adverse events occurring in 10% or more patients

TRAE	250 mg (n=3)		300 mg (n=25)		350 mg (n=53)		Total (n=81)	
	[a] G-All	≥G3	G-All	≥G3	G-All	≥G3	G-All	≥G3
**Gastrointestinal disorders (n, %)**								
**Diarrhea**	3(100)	0	16(64.0)	1 (4.0)	36 (67.9)	1(1.9)	55 (67.9)	2(2.5)
**Oral ulcer**	0	0	4 (16.0)	0	21 (39.6)	1(1.9)	25 (30.9)	1(1.2)
**Vomiting**	1(33.3)	0	8 (32.0)	1 (4.0)	11 (20.8)	0	20 (24.7)	1(1.2)
**Nausea**	1(33.3)	0	8 (32.0)	1(4.0)	7 (13.2)	1(1.9)	16 (19.8)	2(2.5)
**Oral mucositis**	0	0	3 (12.0)	0	7 (13.2)	0	10 (12.3)	0
**Skin and subcutaneous tissue disorders (n, %)**								
**Rash**	1 (33.3)	0	10(40.0)	0	41 (77.4)	7(13.2)	52 (64.2)	7 (8.6)
**Palmar-plantar erythrodysesthesia syndrome**	1 (33.3)	1 (33.3)	7 (28.0)	1 (4.0)	24 (45.3)	2 (3.8)	32 (39.5)	4 (4.9)
**Investigations (n, %)**								
**Elevated AST**	0	0	4 (16.0)	0	17 (32.1)	4 (7.5)	21 (25.9)	4 (4.9)
**Elevated ALT**	0	0	5 (20.0)	0	15 (28.3)	2 (3.8)	20 (24.7)	2 (2.5)
**Leukopenia**	0	0	3 (12.0)	0	6 (11.3)	0	9 (11.1)	0

**Metabolism and nutrition disorders (n, %)**								
**Anorexia**	0	0	2 (8.0)	0	10 (18.9)	1 (1.9)	12 (14.8)	1 (1.2)
**Hypoalbuminemia**	0	0	0	0	12 (22.6)	0	12 (14.8)	0
**Infections and infestations (n, %)**								
**Paronychia**	0	0	4 (16.0)	0	7 (13.2)	1 (1.9)	11 (13.6)	1 (1.2)
**General disorders and administration site conditions (n, %)**								
**Fatigue**	0	0	3 (12.0)	1(4.0)	9 (17.0)	2 (3.8)	12 (14.8)	3 (3.7)
**Blood and lymphatic system disorders (n, %)**								
**Anemia**	1(33.3)	1(33.3)	4 (16.0)	1(4.0)	20 (37.7)	3 (5.7)	25 (30.9)	5 (6.2)
**Renal and urinary disorders (n, %)**								
**Proteinuria**	0	0	3 (12.0)	0	13 (24.5)	0	16 (19.8)	0

[a] G, grade.
